# COVID-19 Resurgence: Lessons Learned to Inform the South African Response

**DOI:** 10.1017/dmp.2021.118

**Published:** 2021-04-19

**Authors:** Mathias Dzobo, Mbuzeleni Hlongwa, Knowledge Denhere, Vincent Kampira, Mathias Mugoni, Godfrey Musuka, Tafadzwa Dzinamarira

**Affiliations:** 1 University of Zimbabwe College of Health Sciences, Harare, Zimbabwe; 2 University of KwaZulu-Natal College of Health Sciences, Durban, South Africa; 3 University of the Western Cape, Bellville, South Africa; 4 ICAP, Columbia University, Harare, Zimbabwe; 5 Optimum Health Medical Laboratories, Gaborone, Botswana

**Keywords:** COVID-19, preparedness, response, resurgence, South Africa

## Abstract

The risk of recurring coronavirus disease (COVID-19) resurgences that threaten Africa’s health care systems, newly opened communities, schools, and businesses looms as communities abandon precautionary measures, such as mask-wearing, physical distancing, and regular handwashing. In this piece, we unpack the handling of both the first wave and subsequent resurgence in the context of 3 countries that are experiencing such a resurgence at the time of writing (December 2020): Israel, France, and the United Kingdom. While it is difficult to extrapolate on what to expect in South Africa, based on experience in these 3 countries, South Africa’s preparedness for a COVID-19 resurgence should place emphasis on the role of expanded testing and isolation capacity, strengthening enforcement of adherence to non-pharmaceutical interventions, and protection of high-risk populations.

## Introduction

In December 2019, a novel coronavirus was identified after a cluster of pneumonia cases of unknown cause were investigated in Wuhan, China. The causative agent was named *severe acute respiratory syndrome coronavirus 2 (SARS-CoV-2)* and is the cause of the coronavirus disease (COVID-19).^1^ Globally, the virus has infected 126.4 million people and caused more than 2.773 million deaths as of March 25, 2021. In Africa, the COVID-19 pandemic has spread to all countries under the World Health Organization (WHO) Africa Region.^2^ South Africa, which constitutes 4.5% of Africa’s population, has reported over 35% (1 541 560) COVID-19 confirmed cases in the region and 47% (52 525) of the deaths in Africa as of March 25, 2021.^2^ The high number of confirmed COVID-19 cases for South Africa has largely been due to the widespread testing conducted in the country compared with other African countries. As of March 25, 2021, South Africa has conducted the highest number of tests (over 9.7 million) and sixth highest number of tests per million population (162 444) in Africa.^3^

The recent months have seen a resurgence of the virus in countries where virus transmission had dwindled to low rates as the first wave of the outbreak had waned. The risk of a resurgence that threatens Africa’s health care systems, newly opened communities, schools, and businesses looms as people abandon precautionary measures, such as mask-wearing, physical distancing, and regular handwashing. In this piece, we unpack the handling of both the first wave and subsequent resurgence in the context of 3 countries that are experiencing such a resurgence at the time of initial writing (December 2020): Israel, France, and the United Kingdom. We discuss measures that South Africa can take to mitigate the impact of a potential COVID-19 resurgence, learning from experiences in the first wave of infections.

**Table 1.** COVID-19 first wave interventions for Israel, France, United Kingdom, and South Africa

	Time of Event	Government Intervention
**ISRAEL**	**(February-May 2020)**	
	21 February 2020	Israel announced a 14-day home isolation rule for anyone who had been in South Korea or Japan.
	19 March 2020	National State of Emergency
	8 April 2020	1800 to 0700 hours curfew
	14-16 April 2020	Partial national lockdown
	4 May 2020	Easing of national lockdown
**FRANCE**	**(February-May 2020)**	
	16 March 2020	The government announced the first lockdown that lasted 15 days.
	13 April 2020	The government extended the lockdown further until May 11.
	11 May 2020	Schools began to reopen as part of the earlier phases of easing the lockdown.
	10 July 2020	State of Health Emergency in France was ended.
	24 July 2020	Compulsory face-mask wearing for all citizens.
**UK**	**(March -May 2020)**	
	3 March 2020	UK Government unveiled their Coronavirus Action Plan.
	23 March 2020	UK first lockdown begins.
	10 May 2020	A roadmap to ease lockdown restrictions is set and people are advised to work from home where possible.
	24 July 2020	Compulsory wearing of face masks was introduced in all public space and indoor venues with fines pegged for those who broke the rule.
**SOUTH AFRICA**	**(April 2020-August 2020)**	
	15 March 2020	National State of disaster was declared by the South African Government.
	27 March 2020	South Africa began its first national lockdown.
	1 May 2020	Phased lifting of lockdown began
	12 July 2020	Extension of the state of disaster due to a rise in COVID-19 cases
	27 July -24 August 2020	Reclosure of all schools to curb the spread of COVID-19.

**Table 2.** COVID-19 second wave interventions in Israel, France, United Kingdom, and South Africa

	Time of Event	Government Intervention
**ISRAEL**	**(September -November 2020)**	
	6 July 2020	A new set of social distancing restrictions amid surging cases of COVID-19
	31 August 2020	“Traffic Light Plan” to indicate the severity of COVID-19 for each city was instituted
	18 September 2020	Government announces a 3-week countrywide lockdown
	18 October 2020	The government eased lockdown restrictions on less severely affected “non-red” cities.
	1 November 2020	Israel eased restrictions further, allowing elementary schools, synagogues, and restaurants.
	20 December 2020	Israel announced an entry ban on all foreign travelers arriving from the United Kingdom, South Africa, and Denmark due to an increase in daily cases caused by new SARS-CoV-2 variants.
	27 December 2020	Israel entered a third national lockdown.
	7 January 2021	Israel declared a 2-week-long, complete lockdown, which was later extended to February 7, 2021.
**FRANCE**	**(August-December 2020)**	
	30 October 2020	Second national lockdown begins in France.
	15 December 2020	Travel restrictions lifted.
	27 December 2020	First person gets Pfizer-BioNTech vaccine.
**UNITED KINGDOM**	**(September-December 2020)**	
	12 October 2020	Three-tier legal framework was introduced to help curb the spread of COVID-19.
	5 November 2020	Beginning of 4-week national lockdown for England that would see pubs, restaurants, leisure centers, and non-essential shops close
	2 December 2020	United Kingdom ended its second lockdown and implemented a replacement 3-tier system.
	2 January 2021	UK became the first country in the world to approve the Pfizer COVID-19 vaccine
	8 January 2021	The first person in the UK was vaccinated.
**SOUTH AFRICA**	**(December 2020-Present)**	
	3 December 2020	The national state of disaster was extended until January 15, 2021.
	28 December 2020	Level 3 partial national lockdown for 3 weeks with a curfew of 9:00 PM–6:00 AM and compulsory wearing of masks
	3 January 2021	Vaccine roll-out commenced.
	28 February 2021	South Africa moves from adjusted level 3 to level 1 as the second wave had passed its peak.

## First Wave Containment, Mitigation, and Easing of Lockdown Regulations

### Israel

**Figure 1.**
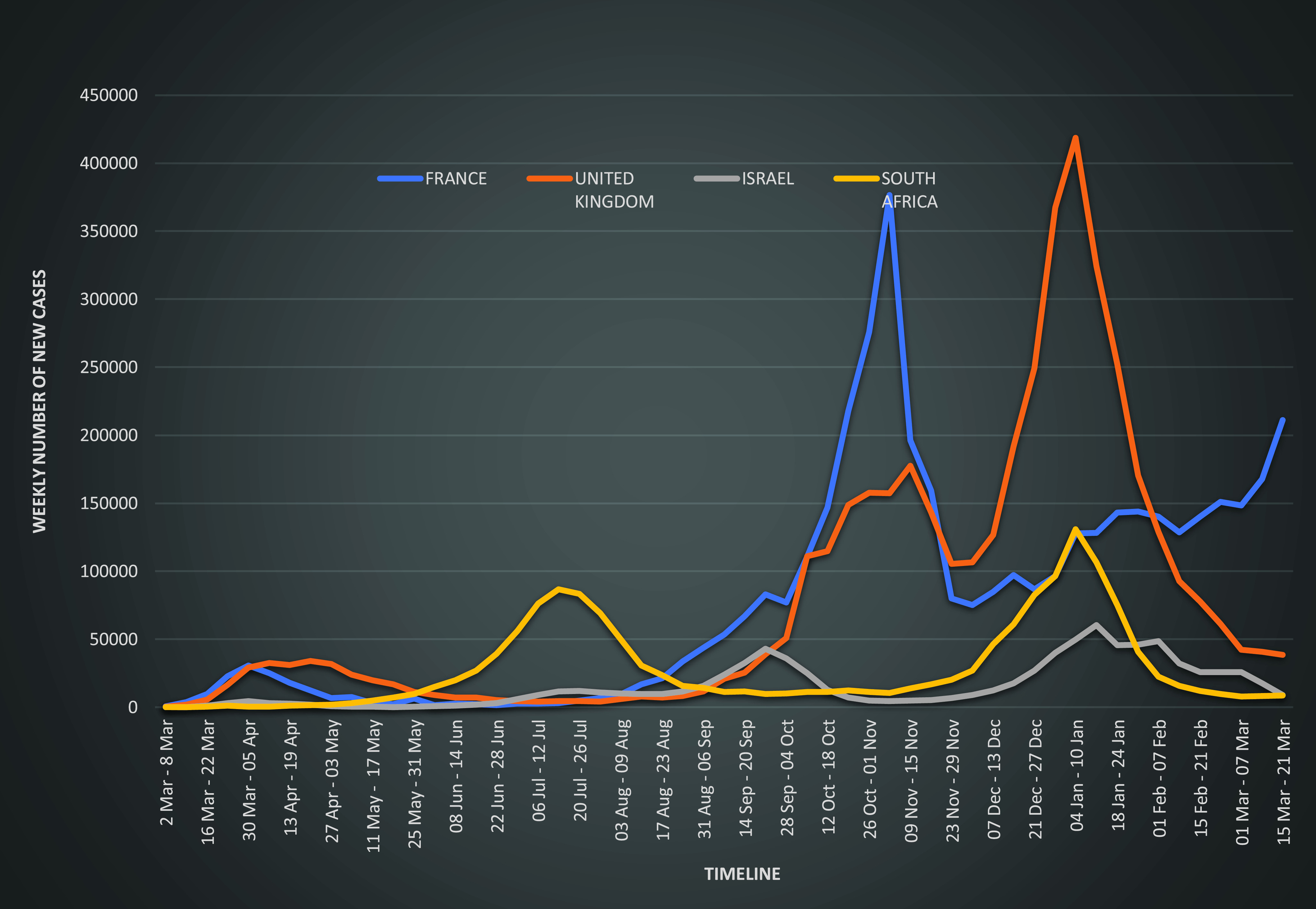
Weekly number of new cases pattern in France, Israel, South Africa, and the United Kingdom.

In Israel, the first infection of COVID-19 was confirmed on February 21, 2020, since the country had reported 35 085 cases and 295 deaths per million population as of November 13, 2020.^4^ Israel’s Ministry of Health (MoH) implemented a raft of containment measures coupled with extensive testing during the early phase of the epidemic. The weekly number of cases pattern for Israel is presented in Figure 1. The containment measures included a 14-day home isolation for people arriving from any country outside Israel and people who had been in contact with a confirmed case of COVID-19. Symptomatic people were instructed to stay home for 2 days after symptom resolution.^5^ On March 11, 2020, gatherings were limited to a maximum of 100 people; this was further restricted to 10 people by March 15, 2020. On March 19, 2020, a national state of emergency was declared in the country, and the first fatality due to COVID-19 was reported on March 20.^6^ Restrictions on the movement of people were introduced with only essential workers and businesses (“essential services”) allowed to move freely. On April 8, 2020, an 1800 to 0700 hours curfew was imposed to prevent people from partaking in joint religious Passover dinners, as per tradition.^7^ More details are presented in Table 1.

On May 4, 2020, the Israeli Government approved a series of amendments to the lockdown regulations that allowed the economy to return to activity. The regulations included a selective return of the education system, retail sector, transportation, and domestic tourism. The government announced a gradual and controlled lockdown relief performed in 4 stages of decreasing strictness that could eventually allow gathering with no limitations. However, at this stage, the government defined one of the following criteria as a cause to return to lockdown: a report of 100 new daily cases of infection (excluding outbreak centers), a viral replication rate of less than 10 days, and 250 patients in a critical condition.^8^

### France

France introduced COVID-19 surveillance on January 10, 2020, 3 days after the identification of the SARS-CoV-2 in China. The first 3 imported cases of COVID-19 in France, the first ones in Europe, were reported 14 days later, on January 24, 2020.^9^ The weekly number of cases pattern for France is presented in Figure 1. As the number of cases rose, the French Government ordered the closure of schools and universities, pubs, restaurants, and cinemas on March 12, 2020. On March 16, 2020, the French President announced the beginning of a lockdown period from March 17, 2020, at noon.^10^ The lockdown period that was earlier planned for 15 days was extended further to May 11, 2020, due to a sharp increase in cases and deaths that took place in early April 2020. The president announced that there would be a gradual lifting of the lockdown after May 11, 2020, with schools and some workplaces reopening but social gatherings and leisure activities would remain banned until at least mid-July 2020. The second phase of the easing of lockdown restrictions was announced on May 28; this saw the previously imposed 100-km radius travel ban being removed and bars/pubs, schools, and restaurants reopening.^11^ On July 10, 2020, the State of Health Emergency in France ended, essentially ending the national lockdown. However, there were still restrictions with emphasis on social distancing and the practice of regular personal hygiene. More details are presented in Table 1.

### United Kingdom (UK)

As cases of COVID-19 rose sharply in Wuhan, China, Public Health England (PHE) announced that it would be moving the risk level to the British public from very low to low on January 22. All passengers aboard flights from Wuhan were screened on arrival at airports as part of the country’s containment measures.^12^ The UK reported its first case of COVID-19 on January 31, 2020, and the country recorded the first death of a person in the UK on March 5, 2020.^4^ The weekly number of cases pattern for the UK is presented in Figure 1. On March 3, 2020, the UK Government launched a 4-pronged action plan with emphasis on keywords *contain*, *delay*, *research*, and *mitigate*. The action plan provided information on the government’s plans to contain the spread of the virus and the action if the virus spreads in the population.^13^ On March 12, 2020, the government shifted from containment to the delay phase, and the government urged people displaying COVID-19 symptoms to self-isolate at home for at least 7 days, no matter how mild their symptoms.^14^ A modeling study conducted by the Imperial College London advised the government to use non-pharmaceutical interventions to reduce the impact of COVID-19-related deaths. These measures included social distancing and isolation of entire households when a family member tested positive, as well as people over age 70 years.^15^ Facing a serious threat of an overwhelmed health system, the UK announced a total lockdown on March 23, 2020, to curb the spread of COVID-19. The government implemented a range of measures, including travel restrictions, social distancing measures, closures of entertainment industry, hospitality, non-essential shops and indoor premises, and increased testing. On May 10, 2020, the government set out a roadmap to ease the lockdown. In the first step, May 13–31, 2020, people were requested to work from home, while those working in the manufacturing and construction sectors were encouraged to go back to work minimizing the use of public transportation. In step 2, from June 1, 2020, some schools and outdoor markets were allowed to reopen, while all other non-essential retail industries reopened on June 15, 2020. In step 3, starting on July 4, 2020, the hospitality and personal care industries, as well as public places, reopened while enforcing social distancing.^16^

## South African Context: Containment, Mitigation, and Easing of Lockdown Regulations

South Africa’s National Institute of Communicable Diseases (NICD) reported its first confirmed COVID-19 case on March 5, 2020.^17^ The weekly number of cases pattern for South Africa is presented in Figure 1. The South African Government declared a national state of disaster on March 16, 2020. A total lockdown came into effect at midnight on March 26, 2020.^16^ Under this declaration, South Africans were urged to observe simple hygiene rules of regular handwashing, social distancing, restricted public gatherings, and meetings to less than 100 people. Restaurants, shops, hubs, churches, and mosques were closed down and there was a ban on the sale of alcohol and tobacco cigarettes. These measures were to curb person-to-person transmission and the spread of the virus.^18^ The South African lockdown was considered one of the most stringent in the continent and possibly globally.

On May 1, 2020, a phased lifting of the lockdown began; the country was moved into level 4, allowing selected sectors to resume operations. On May 13, 2020, a further relaxation of the lockdown was announced, effective June 1, 2020, effectively lowering the restrictions to level 3. Most economic activities reopened under strict health and social distancing practices, except for high-risk ones, for example, entertainment, sporting activities, and conferences. The sale of tobacco remained banned, but the regulated sale of alcohol was allowed. On July 12, 2020, in response to a growing number of COVID-19 cases, a curfew and an alcohol ban were reintroduced and the wearing of face masks in public was made mandatory. The national state of disaster was extended to October 31, 2020. On September 21, 2020, the government scaled down from level 2 to level 1 and eased most of the lockdown restrictions, including international travel to other countries in response to the low number of positive COVID-19 cases.^19^

## COVID-19 Second Wave in Israel, France, and the United Kingdom

Israel, France, and the UK were among the first few countries in the world to respond to a resurgence in COVID-19 cases, interchangeably commonly referred to as a *second wave* of COVID-19. Table 2 presents COVID-19 second wave interventions in Israel, France, United Kingdom and South Africa.

### Israel

Israel has experienced a resurgence of COVID-19 cases since May, when it eased the nationwide lockdown imposed at the start of the pandemic. Israel’s coronavirus cabinet task force approved the “traffic light” plan to tackle the rising COVID-19 infections on August 30, 2020. According to the color-coded system, which commenced on September 6, 2020, restrictions on cities were decided by morbidity rates, green (no restrictions), yellow (minor restrictions), orange (significant restrictions), and red (significant restrictions, including lockdowns).^20^ On September 6, 2020, a 1700 to 0500 hours curfew was imposed on 40 “red zone” communities across the country. On September 10, 2020, Israel became the country with the highest rate of COVID-19 infections per capita.^21^ The virus had infected almost 140 000 of its 9 million population, causing growing criticism of how the prime minister was handling the new wave of infections.^21^ On September 13, 2020, Israel became the first country in the world to impose a second lockdown. The new lockdown measures restricted people to within 500 meters of their homes, except for work and essential activities, such as buying food and pharmacy goods, as well as attending synagogue. On September 23, 2020, a number of cases continued to rise (see Figure 1), Israel imposed a full lockdown over the entire country, with more severe and stricter restrictions than the first lockdown as COVID-19 cases surged. The country’s MoH warned that hospitals were quickly approaching full capacity.

### France

France is facing a second wave of COVID-19, which is more serious than the first. During the country’s first wave, France’s daily new case numbers reached a peak of just over 7500 on March 31, 2020. On October 15, 2020, France became the first country in Europe to record more than 30 000 cases in a day, with 30 621 cases reported.^4^ An 1800 to 0900 hours curfew was imposed in 9 of the country’s largest cities, including Paris. These new regulations came into effect on October 17, 2020. The cities have a combined population of about 20 million people. On October 29, 2020, a second national lockdown was announced.^22^

### United Kingdom

The UK is experiencing some of the highest new daily cases of COVID-19 in the world. The UK reported an average of 14 391 new daily cases in the week of October 12, 2020.^4^ The European Centers for Disease Control and Prevention (ECDC) also reported a growing number of hospitalizations in the UK. In response, the British Government introduced a new 3-tier (medium, high, and very high tiers) system of local COVID-19 alert levels in their varying stages of severity.^23^ The medium tier is a continuation of the measures that are currently in place, such as the rule of 6 and the 2200 hours curfew. Most parts of England are under the medium tier. The high tier will see restrictions that are more stringent on household interactions. The rule of 6 will be maintained at outdoor gatherings. The very high tier restrictions will be applied to areas where infection rates are causing national concern. Social mixing, leisure, and entertainment in these areas will be banned in consultation between local and central governments, but retail, schools, and universities will remain open. There are growing fears that the newly announced 3-tier system may not bring down the rate of infections without the citizens buying into the measures. Several government advisors believe the idea of containing the virus region-by-region is bad for national unity, and instead, they are advocating for a circuit-breaker type of lockdown. A circuit-breaker lockdown would therefore see British citizens stop all contact with people outside their households by shutting non-essential businesses and stopping social interactions. The idea is to interrupt the rate of virus transmissions and allow time for a longer-term plan to be implemented before cases overwhelm the health systems and increase health care worker burnout.^24^

## Lessons Learned to Inform the South African Response/Mitigate the Impact of a COVID-19 Resurgence

South Africa is anticipating another COVID-19 resurgence in the next few months.^25^ In South Africa, the marked increase in new COVID-19 infections (December 2020–February 2021) was associated with the decline in adherence to lockdown restrictions and other measures, such as social distancing, regular sanitizing or handwashing, and mask wearing. In response to COVID-19 resurgences, some countries, such as Israel^20^ and France,^26^ introduced new rounds of curfews and lockdown restrictions to curb the spread of the virus. These public health measures are important to control the transmission of COVID-19. While this is the case, some of the strategies implemented by these countries may not necessarily be as effective in South Africa.

South Africa may attempt to implement curfews and lockdown restrictions to reduce COVID-19 transmission. However, while hard lockdowns managed to slow the rapid spread of COVID-19 at the initial stages, these did not manage to stop the transmission of the virus at the anticipated rates. The reproductive rate of the South African epidemic under various stages of lockdown paradoxically showed a decline as the country went to lower levels of restrictions.^27^ This now suggests less chance of being able to achieve sustained suppression of virus circulation in South Africa through a lockdown, than was the case when circulation initially started.^27^ Second, unlike in UK and France, the timing of the initial lockdown and the likely goals to be achieved differ between the countries. The South African lockdown, unlike in the UK, was not because of impending collapse of health services. It is also worth noting that due to lockdown, many people lost income, while poverty widened^28^ and concerns on a rapid rise of mental health issues arose.^29^ Further hard lockdowns will prove to have dire consequences for the economy.

Following lessons from South Korea, South Africa could mitigate the anticipated COVID-19 resurgence by implementation of robust testing and isolation capacity at community levels. These should be coupled with enforcing strict restrictions on non-pharmaceutical interventions that include mask-wearing, handwashing or sanitizing, social distancing, and reduction of social gatherings. Reinforcement of infrastructure at both public and private health care institutions, as well as protecting high-risk populations, such as elderly and those with comorbidities, remains as important strategies to reduce impact of a COVID-19 resurgence. South Africa recently rolled out a COVID-19 vaccination program.^30^ Efforts to scale up coverage to and ensure high-risk groups are vaccinated before the anticipated surge will further reduce the impact of the anticipated resurgence.

## Conclusion

While it is difficult to extrapolate on what to expect in South Africa, based on experience in Israel, France, or the UK, emphasis should be placed on the role of expanded testing and isolation capacity, strengthening enforcement of adherence to non-pharmaceutical interventions, and protection of high-risk populations.
